# Proteomic Analysis Reveals Physiological Activities of Aβ Peptide for Alzheimer’s Disease

**DOI:** 10.3390/ijms25158336

**Published:** 2024-07-30

**Authors:** Xiaorui Ai, Zeyu Cao, Zhaoru Ma, Qinghuan Liu, Wei Huang, Taolei Sun, Jing Li, Chenxi Yang

**Affiliations:** 1 School of Chemistry, Chemical Engineering and Life Science, Wuhan University of Technology, 122 Luoshi Road, Wuhan 430070, China; aixiaorui@whut.edu.cn (X.A.); czy2023202412@whut.edu.cn (Z.C.); mazhaoru123@whut.edu.cn (Z.M.); liuqinghuan@whut.edu.cn (Q.L.); huangwei2020@whut.edu.cn (W.H.); suntl@whut.edu.cn (T.S.); 2 Hubei Key Laboratory of Nanomedicine for Neurodegenerative Diseases, Wuhan University of Technology, 122 Luoshi Road, Wuhan 430070, China; 3 School of Biological Science & Medical Engineering, Southeast University, No. 2 Sipailou, Nanjing 210096, China

**Keywords:** Alzheimer’s disease, amyloid β, neurodegeneration, mass spectrometry, proteomics

## Abstract

With the rapid progress in deciphering the pathogenesis of Alzheimer’s disease (AD), it has been widely accepted that the accumulation of misfolded amyloid β (Aβ) in the brain could cause the neurodegeneration in AD. Although much evidence demonstrates the neurotoxicity of Aβ, the role of Aβ in the nervous system are complex. However, more comprehensive studies are needed to understand the physiological effect of Aβ_40_ monomers in depth. To explore the physiological mechanism of Aβ, we employed mass spectrometry to investigate the altered proteomic events induced by a lower submicromolar concentration of Aβ. Human neuroblastoma SH-SY5Y cells were exposed to five different concentrations of Aβ_1-40_ monomers and collected at four time points. The proteomic analysis revealed the time–course behavior of proteins involved in biological processes, such as RNA splicing, nuclear transport and protein localization. Further biological studies indicated that Aβ_40_ monomers may activate PI3K/AKT signaling to regulate p-Tau, Ezrin and MAP2. These three proteins are associated with dendritic morphogenesis, neuronal polarity, synaptogenesis, axon establishment and axon elongation. Moreover, Aβ_40_ monomers may regulate their physiological forms by inhibiting the expression of BACE1 and APP via activation of the ERK1/2 pathway. A comprehensive exploration of pathological and physiological mechanisms of Aβ is beneficial for exploring novel treatment.

## 1. Introduction

Alzheimer’s disease (AD) is a neurodegenerative disease that is characterized by progressive cognitive dysfunction and behavioral damage. Currently, more than four million people in the world are suffering from dementia, and 60–70% of cases are diagnosed with AD [1,2]. However, only palliative treatment is currently available for AD patients. The demand for developing effective therapies requires advances in the understanding of AD pathogenesis. The pathological deposition of amyloid-β peptide (Aβ), which is known as senile plaques (SPs), is usually found in AD [3,4]. Aβ is a small peptide with 40–42 amino acids and is derived from the cleavage of amyloid precursor protein (APP) by the β-site secretase enzyme (BACE-1) and γ-secretases [5,6,7]. The structures of Aβ in AD include monomers, small soluble oligomers, large fibrils and plaques [8,9,10]. In the case of AD, many believe that Aβ is associated with degenerating neurons [11]. Aβ predisposes the cultured neurons to die via mechanisms that include oxidative stress and a disruption of cellular calcium homeostasis [12]. The oxytosis-/ferroptosis-regulated cell death pathway is also reported to be affected by intracellular Aβ [13].

Although much evidence demonstrates the neurotoxicity of Aβ [14], the roles of Aβ in the nervous system are complex. Recent studies have revealed that the inhibition of APP metabolism can affect the viability of cortical neurons due to the decline in the neuronal Aβ level [15]. Several studies have suggested that the secretion of Aβ can regulate synaptic plasticity and maintain a physiological homeostasis for neuronal activity [16,17]. Those studies have implied a key physiological role for the Aβ peptide. Aβ is recognized to be linked to neuronal degeneration due to its direct cellular toxicity and the toxic compounds generated from a pronounced inflammatory response. Therefore, a greater understanding on the physiological function of Aβ may shed light on the mechanisms involved in the neurotoxicity of Aβ in AD. The biological mechanisms regulated by Aβ at physiological concentrations remain unknown.

Over the past two decades, mass spectrometry (MS), with its increasing power of comprehensively profiling proteome, has been successfully applied to decipher biological mechanisms and pathogenesis of diseases [18,19]. Herein, we employed mass spectrometry to investigate the altered proteomic events stimulated by Aβ_1-40_ with the aim to investigate the physiological mechanism of Aβ in vitro. Human neuroblastoma SH-SY5Y cells treated with five different concentrations of Aβ_1-40_ were collected at different times. MS analysis revealed more than 300 significantly changed proteins. Further Gene Ontology analysis indicated the time–course behavior of proteins induced by Aβ_1-40_. Subsequent biological studies indicated that the pathways of glycogen synthase kinase-3β (GSK-3β) and AKT are involved in the regulation of the physiological forms of Aβ. Moreover, MAP2, p-Tau and Ezrin play important roles in modulating the cytoskeleton of neuron cells and neuronal differentiation. Our evidence has suggested that Aβ_40_ monomers may regulate their physiological forms by inhibiting the expression of BACE1 and APP via activation of the ERK1/2 pathway. These results implicated that Aβ_40_ monomers may inhibit the activities of Aβ synthesis enzymes to reduce Aβ productions via the negative feedback, which is line with the “loss-of-function” hypothesis of Aβ_40_ monomers in AD.

## 2. Results

### 2.1. Overview of Proteomic Analysis for Aβ-Induced SHY5Y Cells

Aβ production has been found to affect the viability of cortical neurons. Most studies have focused on Aβ toxicity while the physiological activities of Aβ monomers in the metabolism remain unknown. The physiological concentration of Aβ is estimated to be less than 1 μM. When the concentration exceeds 3 µM, Aβ is microscopically visible in monomeric form [17]. Although previous studies have investigated the effects of Aβ at a concentration below 1 µM on neuronal death, these studies primarily focused on the observation of cell viability [18]. The underlying biological events triggered by the physiological concentration of Aβ are still unclear. Herein, a comprehensive proteomic analysis was conducted to study the proteome affected by low concentration of Aβ peptide.

**Figure 1:**
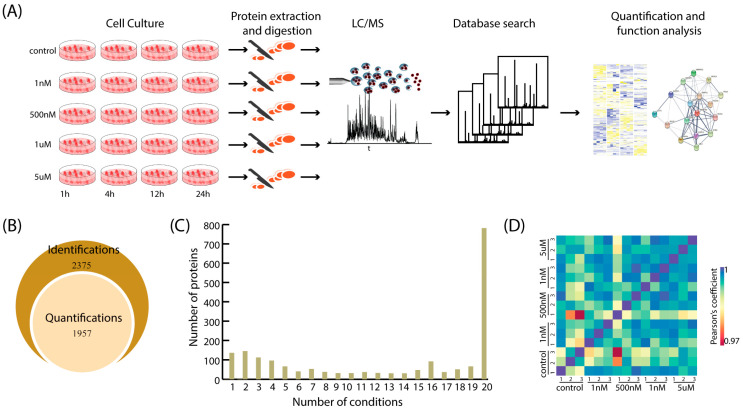
Characterization of Aβ induced proteome by mass spectrometry. (**A**) The workflow of proteomic analysis. SHY5Y cells were treated with 1 nM, 500 nM, 1uM and 5 uM of Aβ_1-40_ and then collected at 1 h, 4 h, 12 h and 24 h. Proteins were extracted from each sample and digested before LC/MS analysis on a Q-Extractive Plus mass spectrometry. Protein identification was performed by searching MaxQuant. Perseus was used for statistical analysis to reveal differential expressed proteins. DAVID and STRING were employed for further function analysis. (**B**) Stacked Venn diagram showing the fractions of identified and quantified proteins from the samples in all conditions. (**C**) Histogram representing the number of quantified proteins across all conditions. (**D**) Heatmap of Pearson’s coefficient for samples collected at 1h. Samples in each condition were prepared in triplicates. A heatmap of Pearson’s coefficient for samples at all conditions is shown in Figure S1.

To investigate the effect of low concentrations of Aβ peptides on the proteome of SH-SY5Y cells, four different concentrations of aggregated Aβ_1-40_, 1 nM, 500 nM, 1 µM and 5 µM, were added to the cells. Previous studies have suggested that the expression of the immediate-early genes and late-response genes occurs on a time scale of hours during stimulation [20]. SH-SY5Y cells were, therefore, collected after an incubation of Aβ for 1 h (h), 4 h, 12 h and 24 h (Figure 1A). To profile the proteome induced by Aβ_1-40_, proteins were extracted from each sample, digested and, analyzed individually using LC/MS. Two or three biological replicates were prepared for each condition, and each biological sample was analyzed twice with MS. A total of 16,751 unique peptides derived from 2375 proteins were identified by searching against a human proteome database (downloaded from UniProt which contains 202,160 entries) in MaxQuant software, v1.6.17.0 (Tables S1 and S2). On average, about 9500 unique peptides and 1500 unique proteins were identified from each biological sample in a single MS analysis. Proteins identified with at least two unique peptides and in at least two conditions at the same time point were considered for quantification analysis. Among the 2375 identified proteins, 1957 proteins (~82.4%) were quantified across all conditions (Figure 1B). As shown in Figure 1C, the majority of proteins (1239 out of 1957) were quantified in at least ten different conditions. About 40% of proteins (782 out of 1957) were quantified in all conditions. To further determine the quality of our proteome analysis, Pearson correlation was performed. Between biological replicates of the cells collected at 1 h, Pearson’s correlation coefficients were about 0.98 which demonstrates the good technical and biological reproducibility of our MS data (Figure 1D).

### 2.2. General Insights into Differential Expressed Proteins Identified with MS

**Figure 2:**
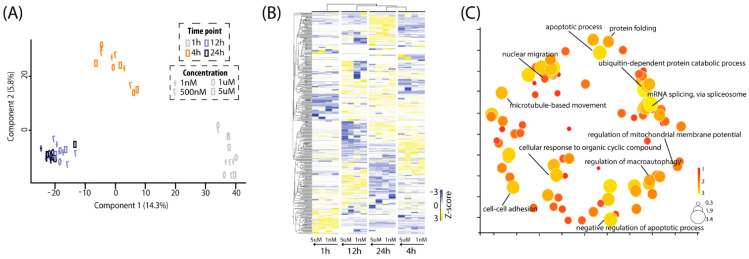
Quantification analysis of Aβ-induced proteome changes from mass spectrometry. (**A**) PCA for all quantifiable proteins (n = 1957) showed the effects of Aβ concentration (component 2 axis) and treatment duration (component 1 axis) on the proteomes of SHY5Y cells. Control samples at each time point were used for normalization in order to compare the changes of protein expression across all conditions. Color: time point; Symbol: concentration. (**B**) Heatmap for differentially expressed proteins (n = 389) from all conditions. The data from control samples were served as the basal change of proteome at their corresponding time point. The Z-score was calculated by mean normalization of the log2 fold change of the differentially expressed proteins. (**C**) Biological process analysis (*p* < 0.05) from DAVID for all the differential expressed proteins and visualization using REViGO. In the bubble chart, the GO annotation for the differentially expressed proteins were presented according to semantic similarity. Size of circles is proportional to the number of the annotated GO terms.

To gain an overview of the proteome of SH-SY5Y cells stimulated by Aβ_1-40_, a principal component analysis (PCA) of all quantified proteins across all conditions was performed (Figure 2A). As depicted in Figure 2A, the component 1 axis clearly illustrated the time–course behaviors of the proteomes in SHY5Y cells from 1 h to 12 h, which were more similar at 12 h and 24 h. The component 2 axis indicated the degree of similarity among the different concentrations of Aβ_1-40_ treated cells at each time point. The distances of data points from 1 nM, 500 nM, 1 μM and 5 μM were relatively close at each time point. These results suggested that the proteome induced by different low concentrations of Aβ exhibited similar behavior at the same time point while the treatment duration had more significant effects on the proteomes from 1 h to 12 h.

After being filtered by fold changes and statistical analysis, 389 differentially expressed proteins were identified (Table S3). A heatmap was generated to demonstrate the distinct protein expression patterns across the samples under different conditions (Figure 2B). Hierarchical clustering analysis of these differentially expressed proteins showed that the hierarchical matrix was divided into four groups according to the treatment duration. This co-segregation suggested that the response of SH-SY5Y cells to different concentrations of Aβ was similar for same treatment duration, which is consistent with the results of PCA. To understand the functional roles of these differentially expressed proteins, DAVID analysis was conducted. The annotation results were then submitted to REViGO to reduce redundant terms and display them based on their similarity and relationships (Figure 2C) [21]. Accordingly, the enriched annotations were able to be categorized as follows (Table S4): (1) mRNA splicing; (2) nuclear migration and protein localization, including biological processes like mRNA export from nucleus, endoplasmic reticulum to Golgi vesicle-mediated transport; (3) cell–cell adhesion; (4) nucleus and endosome organization, such as nuclear envelope organization, multivesicular body assembly; (5) cytoskeleton organization, such as mitotic spindle organization, mitotic cell cycle; (6) apoptotic process; (7) ubiquitin-dependent protein catabolic process.

### 2.3. Time–Course Pattern Analysis of Aβ-Induced Proteomic Events

**Figure 3:**
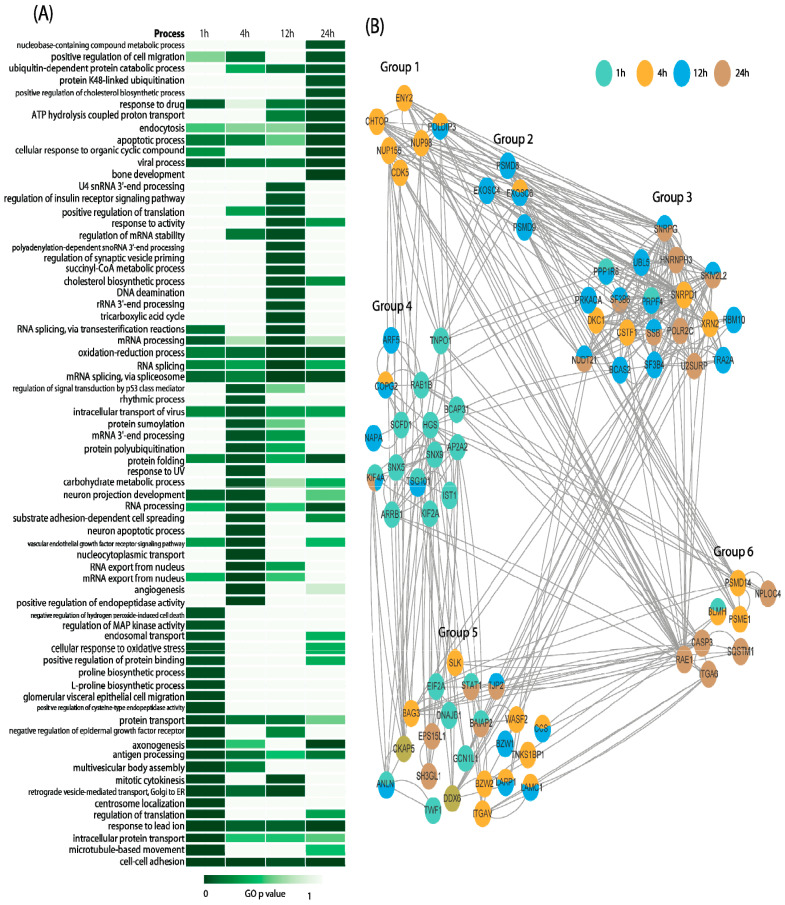
GO and PPI analysis for differentially expressed proteins. (**A**) Heatmap for functional annotation of differentially expressed proteins found at each time point; (**B**) STRING analysis for selected differential expressed proteins at each time point. Group 1: nuclear transport; Group 2: RNA metabolic process; Group 3: RNA splicing; Group 4: protein localization; Group 5: cell adhesion; Group 6: ubiquitination and cellular response to organic cyclic compound.

As demonstrated above, the behavior of the proteome induced by different concentrations of Aβ was more similar compared to the changes that varied according to the duration of Aβ treatment. Therefore, we firstly investigated the time–course patterns of proteomic events induced by Aβ. The lists of differentially expressed proteins identified at each time point were submitted separately to DAVID for functional enrichment annotation. To better illustrate the data, a heatmap for Gene Ontology (GO) analysis was created as shown in Figure 3A. We further explored potential associations among these patterns with PPI analysis through STRING (Figure 3B).

As shown in Figure 3B, the expression levels of proteins involved in cell–cell adhesion were affected by the incubation duration with Aβ, ranging from 1 h to 24 h, in comparison to control samples (*p* value < 0.05) (Group 5 in Figure 3B). At 1 h, the alter changes of proteins were mainly related to protein localization (Group 4 in Figure 3B). Meanwhile, the majority of proteins associated with nuclear transport showed significant changes after being induced by Aβ for 4 h (Group 1 in Figure 3B). With the increasing duration of treatment, proteins involved in RNA metabolism, especially regulation of RNA splicing, were dramatically affected by Aβ after 12 h (Group 2 and 3 in Figure 3B). Moreover, proteins with significant changes at 24 h were mainly related to ubiquitination and a cellular response to organic cyclic compounds (Group 6).

### 2.4. Physiological Pathways Stimulated by Aβ Monomers in Neuronal Cells

**Figure 4:**
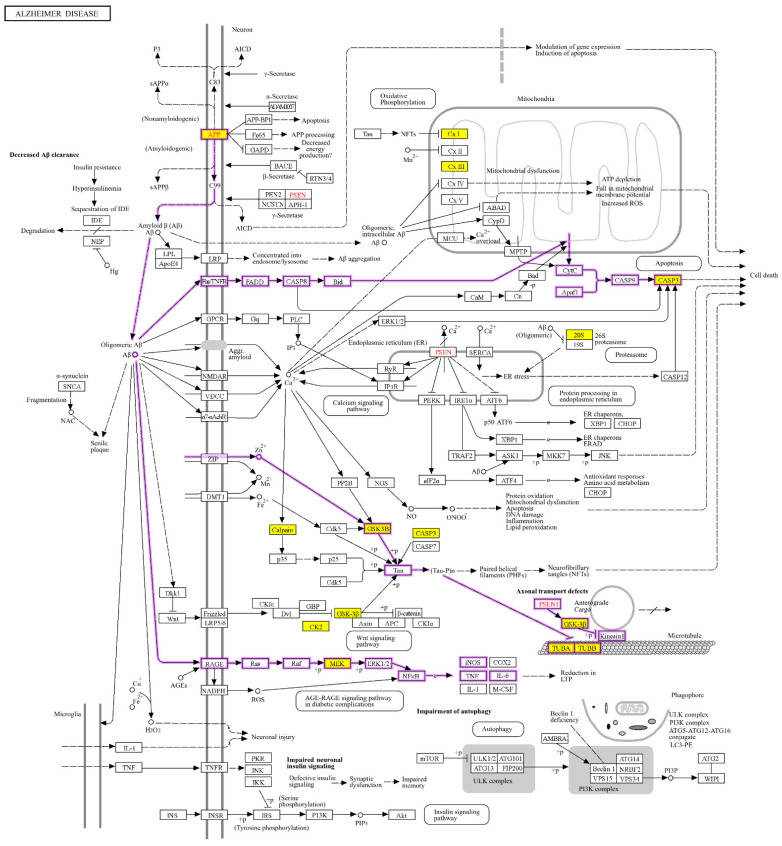
KEGG analysis for differentially expressed proteins identified by LC/MS and results for enriched pathways associated with AD. Yellow boxes: regulated genes; Purple circuit diagram: possible signal pathways.

KEGG analysis was further performed for differentially expressed proteins identified with MS. Results indicated that multiple proteins are associated with neurodegeneration (Figure 4). As shown in Figure 4, the significantly enriched pathways affected by Aβ_40_ include the MAPK pathway, insulin pathway and Ras pathway. Proteins enriched in these pathways can be classified as signal molecules such as GSK-3β, Aβ metabolism regulators such as APP and proteins related to the axon and dendrite of neurons such as Tau. In the following studies, we focused on three signal molecules, AKT, ERK and GSK-3β, to further investigate the impact of Aβ monomers on physiological pathways. The expression of BECA1 and APP was also examined to understand the metabolism of Aβ. Furthermore, the effect of Aβ monomers on key elements, such as Tau, Ezrin and MAP2, that are linked to the axon and dendrite of neurons was explored.

### 2.5. Aβ Regulating ERK, AKT and GSK-3β through Their Phosphorylation

As shown in Figure S2, when the concentration of Aβ was lower than 5 µM, it did not exhibit toxicity. This phenomenon may explain the similar proteomic behaviors shown in the above MS studies. However, MS results indicated that proteins involved in the apoptosis process were significant changes even at 1 nM of Aβ (Figure 3A). Previous studies have demonstrated the crucial role of ERK phosphorylation in modulating the cell’s survival and apoptosis [22,23]. Moreover, the increasing phosphorylation levels on Y216 (tyrosine 216) from GSK-3β was observed in degenerating cortical neurons induced by ischemia [24]. We, therefore, hypothesized that protein phosphorylation was the alternative approach for Aβ to regulate the physiological pathways. This may explain the results presented in Figure 4 that demonstrate that differential expression was found in GSK-3β and MEK, one of proteins in the upstream process of ERK pathway, while no significant changes were revealed in other pathways, such as the insulin pathway. AKT in the insulin pathway could inhibit the serine phosphorylation on GSK-3β to facilitate keeping the balance between insulin receptors and synaptic activity, thereby enhancing the cognition [25,26].

**Figure 5:**
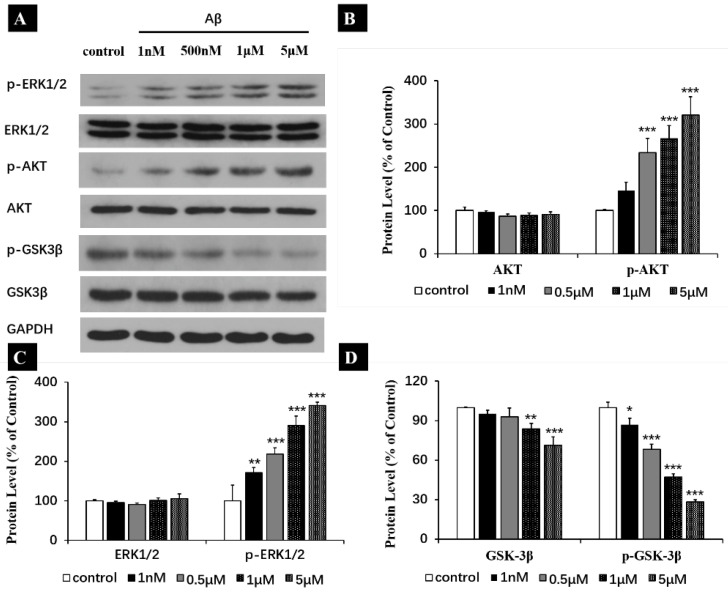
Effect of Aβ (1 h) treatment on the expression of AKT, p-AKT, ERK1/2, p-ERK1/2, GSK-3β and Y216 p-GSK-3β in the human neuroblastoma SH-SY5Y cell line. (**A**) Representative western blot and densitometry analysis of AKT, p-AKT, ERK1/2, p-ERK1/2, GSK-3β and p-GSK-3β. (**B**–**D**) Western blot analysis for AKT, p-AKT, ERK1/2, p-ERK1/2, GSK-3β and p-GSK-3β. All data are presented as mean ± SEM. * *p* < 0.05, ** *p* < 0.01 and *** *p* < 0.001, compared to control.

As shown in Figure 5A–C, incubation of Aβ monomers for 1 h led to the increased phosphorylation levels on ERK1/2 (increased by 71 ± 4.5% for 1 nM, 118 ± 5.4% for 0.5 μM, 190 ± 8.0% for 1 μM, 241 ± 2.7% for 5 μM) and AKT (increased by 46.3 ± 6.2% for 1 nM, 134.0 ± 11.0% for 0.5 μM, 165.8 ± 5.9% for 1 μM, 220.8 ± 14.1% for 5 μM). These results indicated that Aβ monomers activated ERK1/2 and an AKT pathway through regulating their phosphorylation levels, which have supported our previous hypothesis. Further studies showed that Aβ monomers not only decreased the total expression levels of GSK-3β (4.8 ± 2.8% at 1 nM, 7.1 ± 6.7% at 0.5 μM, 16.1 ± 4.1% at 1 μM and 28.8 ± 6.5% at 5 μM), but also inhibited the phosphorylation levels of GSK-3β on Y216 (13.1 ± 4.9% at 1 nM, 21.7 ± 3.7% at 0.5 μM, 53.0 ± 2.7% at 1 μM and 71.8 ± 1.6% at 5 μM) (Figure 5A,D). These results revealed that Aβ monomers reduced the total expression of GSK-3β and decreased the activity of GSK-3β by inhibiting phosphorylation of Y216.

### 2.6. Aβ Monomers Inhibiting the Expression of APP, BACE1 and p-Tau to Achieve Their Physiological Functions

As mentioned earlier, the behaviors of Aβ under physiological conditions are different compared to those under pathological conditions [15,27,28,29,30,31,32]. In contrast to its role in AD, Aβ at picomolar concentration could rescue the viability of cortical neurons affected by the inhibition of the two enzymes (β- or γ-secretase) [15]. It has also been reported that synthetic Aβ_40_ at picomolar concentrations are able to enhance synaptic plasticity and memory in the hippocampus [29]. In order to look into the roles of Aβ under physiological conditions, we put our emphasis on the processes of Aβ production and regulation of the axon and dendrite of neurons. After incubation with Aβ, western blot (WB) analyses were conducted for APP, BACE1 and p-Tau. Because APP could generate Aβ through endoproteolytic cleavage and was found differentially expressed in MS data. BACE1 is another Aβ production-related protein. BACE1 knockout mice exhibit behavioral deficits and synaptic dysfunction [28]. Moreover, many studies suggest that Tau is an axonal marker located in the soma and axons of neurons [30].

**Figure 6:**
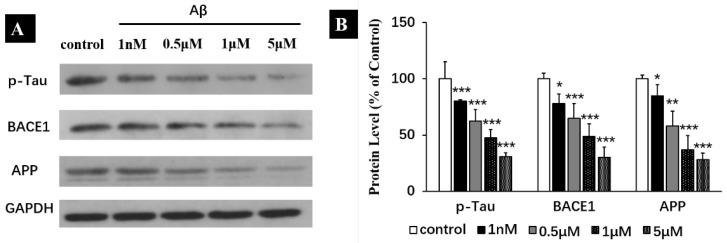
Effect of Aβ (1 h) treatment on the expression of p-Tau, BECA1 and APP in the human neuroblastoma SH-SY5Y cell line. (**A**) Representative western blot and densitometry analysis of p-Tau, BECA1 and APP. (**B**) Western blot analysis for p-Tau, BECA1 and APP. All data are presented as mean ± SEM. * *p* < 0.05, ** < 0.01 and *** *p* < 0.001, compared with control.

The results in Figure 6 revealed that treatment with Aβ monomers for 1 h significantly decreased the expression of APP (by 15 ± 9.8% for 1 nM, 41.5 ± 13.4% for 0.5 μM, 62.3 ± 12.3% for 1 μM and 71.2 ± 5.8% for 5 μM) and BECA1 (22 ± 8.5% for 1 nM, 35.1 ± 13.1% for 0.5 μM, 51.2 ± 11.1% for 1 μM and 69.8 ± 9.2% for 5 μM). These proved that the acute increase of Aβ_40_ levels decreased the expression of BECA1 and APP which may implicate the decreasing Aβ production. Additionally, one hour of Aβ treatment also inhibited the phosphorylation levels of Tau (S404) (decreased by 20 ± 1.5% for 1 nM, 37.5 ± 10% for 0.5 μM, 52.2 ± 7.5% for 1 μM and 69.1 ± 3.2% for 5 μM) in a dose-dependent manner (Figure 6A,B). Unlike hyperphosphorylation in Tau reported previously in AD model [32], our results found that Aβ monomers decreased the phosphorylation levels of Tau on Ser404, which is consistent with the physiological effect of Aβ.

### 2.7. Prolonged Exposure of Aβ Monomers Increasing the Expression of MAP2, Ezrin and APP

The loss of microtubule-associated proteins (MAPs) was observed in MS results when SH-SY5Y cells were exposed to Aβ for 1 h (Table S3). However, the accumulation of Aβ aggregations and MAP2 are observed in the stratum lacunosum moleculare [33]. MAP2 usually serves as a differentiation marker for mature neurons in the late-stage neural differentiation [34]. In addition to MAP2, Ezrin is another crucial protein linked to neuronal differentiation. Reduced Ezrin in HTLA-230 cells could result in the increased activity on proliferation and migration, but it produces a loss of differentiation in cell morphology [35].

**Figure 7:**
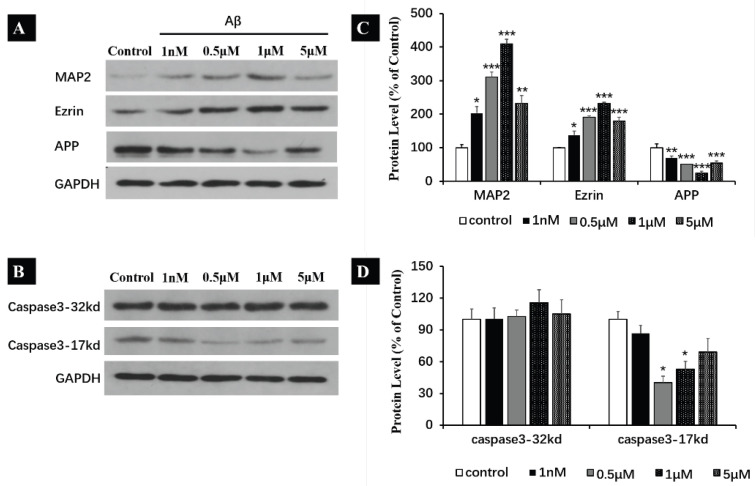
Effect of Aβ_40_ monomers (24 h) treatment on the expression of MAP2, Ezrin, APP, caspase-3-32kd and caspase3-17kd in the human neuroblastoma SH-SY5Y cell line. (**A**,**B**) Representative western blot and densitometry analysis of MAP2, Ezrin, APP, caspase-3-32kd and caspase3-17kd. (**C**,**D**) Western blot analysis of Ezrin, APP, caspase-3-32kd and caspase3-17kd. All data are presented as mean ± SEM. * *p* < 0.05, ** *p* < 0.01 and *** *p* < 0.001, compared with control.

Herein, we investigated the expression levels of MAP2 and Ezrin in SH-SY5Y cells with prolonged exposure to Aβ monomers (24 h). The expression of MAP2 exhibited an increase of 103.2 ± 19.1% at 1nM, 210.3 ± 25.3% at 0.5 μM, 308.8 ± 14.6% at 1 μM and 131.8 ± 23.9% at 5 μM (Figure 7A,C). In Figure 7A,C, a notable increase in the expression of Ezrin was revealed (increased by 37.6 ± 12.1% at 1 nM, 100 ± 28.2% at 0.5 μM, 131.7 ± 3.7% at 1 μM and 79.4 ± 10.9% at 5 μM). These suggest that Aβ monomers may promote the differentiation of neuroblastoma SH-SY5Y through activating MAP2 and Ezrin.

With the prolonged exposure to Aβ, the expression of proteins enriched in the apoptotic process was significantly changed, such as caspase-3 (CASP3), as illustrated in Figure 3A and Figure 4. Researchers have discovered that caspases or caspase-like proteases can cleave APP to release a C-terminal-derived peptide. This C-terminal peptide has 31 amino acids and has been shown to exhibit cytotoxic effects in cultured neurons. Furthermore, in APP-overexpressing transgenic mice, an increase in caspase-3 activity was observed at the onset of memory impairment [36].

Accordingly, western blot analyses were performed to evaluate the expression of caspase-3 and APP when SH-SY5Y cells were incubated with Aβ for 24 h. A reducing expression of APP was detected with western blot (decreased by 29.1 ± 5.3% at 1 nM, 48.9 ± 4.8% at 0.5 μM, 75.3 ± 5.3% at 1 μM and 45.8 ± 6.9% at 5 μM compared to the control) (Figure 7A,C). Although no obvious change was detected on the 32kD caspase-3 precursor, the 17kD caspase-3 exhibited a decreased expression (13.3 ± 7.7% at 1 nM, 59.9 ± 6.0% at 0.5 μM, 47.1 ± 7.4% at 1 μM, 31 ± 12.8% at 5 μM compared to the control) (Figure 7B,D). These findings have demonstrated that Aβ_40_ monomers inhibit the expression of APP and caspase-3, which suggests a potential physiological mechanism at play.

## 3. Discussion

The amyloid hypothesis for AD is based on the observation that senile plaques are composed mainly of Aβ (40 or 42) proteins [3,4]. Aβ monomers can form various structures of aggregations including oligomers, protofibrils and amyloid fibrils. NMR-guided simulations of Aβ_40_ and Aβ_42_ suggested that Aβ_42_ has a great propensity to form amyloid deposits in an AD model compared to Aβ_40_. However, about 80–90% of Aβ in the biological system exists in the form of Aβ_40_ [37]. Recent studies have identified the importance of small oligomers for Aβ toxicity. Meanwhile, Aβ monomers have proved to be the products of cellular metabolism and have neuroprotective effect. The questions on identifying the physiological and pathologic forms of Aβ and deciphering the role of Aβ in dementia still need to be addressed. Therefore, we performed the proteomic studies to investigate the physiological effect of Aβ_40_ monomers under physiological conditions.

The differentially expressed proteins identified in the Aβ_40_-treated SH-SY5Y cells are generally required for maintaining basic cellular activities. DAVID analysis showed that the biological processes induced by Aβ_40_, such as cytoskeleton organization, cell–cell adhesion and apoptotic processes, could influence neuronal survival and physiological growth. Further KEGG analysis revealed that Aβ_40_ may regulate the PI3K-AKT signaling pathway, the MAPK signaling pathway, the mTOR signaling pathway, the insulin signaling pathway and the Ras signaling pathway. The subsequent western blot analyses have demonstrated that AKT and ERK1/2 could be activated by Aβ_40_ monomers. Interestingly, we found that Aβ_40_ monomers could modulate the pathways of ERK1/2 and PI-3-K through increasing the phosphorylation levels of AKT and ERK1/2, whereas Aβ_42_ monomers have only been reported to activate the PI-3-K pathway [38]. Moreover, our studies indicated that Aβ_40_ monomers decreased the expression of BECA1 and APP. These results implicated that Aβ_40_ monomers may inhibit the activities of Aβ synthesis enzymes to reduce Aβ productions via the negative feedback, which is consistent with the “loss-of-function” hypothesis of Aβ_40_ monomers in AD. It is worth noting that Aβ_40_ monomers inhibited the Ser404 phosphorylation of Tau in our studies, whereas the increasing phosphorylation levels on Ser396/Ser404 from Tau have been reported to result in synaptic failure in AD [39,40]. It is, therefore, reasonable to speculate that Aβ_40_ monomers decreased the phosphorylation of Tau to maintain the normal neuronal and synaptic functions.

All the studies presented here, taken together, indicate that Aβ_40_ monomers may activate PI3K/AKT signaling to regulate p-Tau, Ezrin and MAP2. It must be point out that Aβ_40_ monomers have the ability to inhibit the expression of BACE1 and APP via the ERK1/2 pathway. However, more comprehensive studies are needed to understand the physiological effect of Aβ_40_ monomers in depth.

## 4. Materials and Methods

### 4.1. Sample Preparation of Aβ_40_ Monomers Solution

Lyophilized Aβ_40_ powder (1 mg package) was separately dissolved in 1 mL HFIP (Sigma, Shanghai, China, Cat# 52517). Then, the mixed solution of HFIP and Aβ_40_ peptides was vortexed in an ice bath for 3 h at moderate speed. Subsequently, the mixture was dried under a gentle stream of high-purity nitrogen gas for 50 min. The dried Aβ_40_ monomer powder was completely dissolved in 200 μL dimethyl sulfoxide (DMSO) (Gibco, New York, NY, USA, Cat# D12345) to yield a 1150 μM stock solution and stored at −80 °C. The freshly prepared Aβ_40_ peptides were used at the appropriate concentration.

### 4.2. Cell Culture and Aβ Treatment

The human neuroblastoma SH-SY5Y cell line was purchased from American Type Culture Collection (Rockville, MD, USA). The cells were cultured in Dulbecco’s Modified Eagle Medium (MEM) (MEM, Gibco, Cat# C12571500BT) supplemented with 10% fetal bovine serum (FBS, Gibco, Cat# A3160802) and 1% penicillin/streptomycin (Gibco, Cat# 15140122). All cells were cultured at 37 °C under 5% CO_2_. For Aβ treatment, cells of logarithmic growth phase were seeded in six-well plates (6 × 10^5^ cells/well). After cultured for 24 h, the cells were divided into five groups (n = 3 samples/group): control (DMSO), 1 nM, 500 nM, 1 μM, 5 μM of Aβ treatment for 1 h, 4 h, 12 h and 24 h. After incubation, the protein was harvested and ready for use. The cell viability was detected with CCK-8 assay according to the instructions given by manufacturer (Dojindo Molecular Technologies, Gaithersburg, MD, USA).

### 4.3. Protein Extraction and Digestion

Cells were harvested and frozen in −80 °C before use. For protein extraction, twice volume of lysis buffer (8 M urea, 40 mM NaCl, 5 mM CaCl_2_, 100 mM Tris, pH ≈ 8) were added into one volume of cell pellets. The total protein concentration of each sample was measured using a BCA kit (Pierce, Rockford, IL, USA). Disulfide bonds were reduced with 5 mM dithiothreitol (DTT) for 45 min at 37 °C and alkylated with 15 mM iodoacetamide (IAA) for 30 min at dark, which was quenched by 5 mM DTT for 15 min at room temperature. The samples were diluted with 100 mM Tris (pH ≈ 8) until the concentration of urea was below 1 M. Trypsin (Promega, Madison, WI, USA) was added into samples with the protein-to-enzyme mass ratio of 50:1, and samples were incubated in 37 ℃ water bath overnight. The digested samples were desalted with Sep-Pak C18 column (Waters, Milford, MA, USA) and dried by vacuum before LC/MS analysis.

### 4.4. MS Analysis

All MS analyses were conducted on a Q Exactive Plus mass spectrometer (Thermo Fisher Scientific, Waltham, MA, USA). For each analysis, 3 μL of samples were separated by an EasyLC 1000 (Thermo Fisher Scientific, Waltham, MA, USA) using C18 nano column. The LC gradient was increased from 5% to 35% B in 120 min (mobile phase A: 0.1% formic acid (FA) in water; mobile phase B: 0.1% FA in ACN) at flow rate of 0.3 ul/min. MS was operated in data-dependent mode in which the twenty most abundant ions in MS1 were selected for MS2 analysis. The resolution of 70,000 and an AGC target of 3E6 were used for MS1 analysis. The scan range was from 300 to 2000 *m*/*z*. For MS2, resolution of 17,500 and AGC target of 1E5 were selected. The dynamic range was 60 s and max. Injection times were 200 ms for MS1 and 100 ms for MS2. The normalized collisional energy was 30%.

### 4.5. MS Data Analysis

The MS data was processed using MaxQuant with the Homo Sapiens database downloaded from UniProt (71,544 entries). Default settings were used for the MaxQuant search. Briefly, carbamidomethylation of cysteines was set as fixed modification, whereas variable modifications were set as oxidation of methionine and N-terminal acetylation. Trypsin was selected for protein digestion with maximum three miss cleavage sites. The FDRs of 1% were applied for peptide and protein identification.

A setting of label-free quantification in MaxQuant was applied for quantification analysis. Match between run was also enabled with default setting. The statistical analyses were conducted with Perseus. LFQ intensity of proteins were logarithm transform, and miss values were replaced from normal distribution using the Imputation function of Perseus with width of 0.3 and down shift of 1.8. Only the proteins with at least two unique peptides and identified in more than two biological samples at the same time point were considered for quantification analysis. The proteins which satisfied one of the following criteria were selected for function analysis: (1) proteins with 1.5 fold changes and *p* value ≤ 0.05 (one-way ANOVA); (2) proteins only identified in control samples while missing in the corresponding treated samples and their corresponding log1.5 (treatment/control) ≤ −4; (3) proteins only identified in certain treatment groups while missing in the corresponding control samples and their corresponding log1.5 (treatment/control) ≥ 4. Function analyses were performed with the Gene Ontology (GO) and Kyoto Encyclopedia of Genes and Genomes (KEGG) pathway annotation using DAVID online tools. The total identified proteins from all groups (or the entire human dataset) were uploaded onto DAVID as background for calculation of ontology enrichment. A corrected *p* value < 0.05 is considered as significant in GO analysis. STRING was employed to perform protein–protein interaction network analysis (PPI) [41]. MS data were deposited to the ProteomeXchange Consortium via the MassIVE with the data set identifier PXD053527 and PXD053519.

### 4.6. Western Blot Validation

The SH-SY5Y cells were homogenized in RIPA cell lysis buffer containing a protease inhibitor cocktail and phenylmethanesulfonyl fluoride (Beyotime, Wuhan, China). The protein concentration was detected by BCA protein assay (Beyotime, Wuhan, China, Cat. No. P0010). First, 10–40 μg proteins were loaded onto 5–8% gels (Bio-Rad Laboratories, Shanghai, China) and run for 1.5 h at 120 V. The proteins were then transferred to polyvinylidene difluoride (Merck Millipore, Billerica, MA, USA, #IPVH00010) membranes at 100 V for 1 h. The membranes were blocked in 5% non-fat milk for 2 h in tris-buffered saline with 0.1% Tween 20 (TBST) at room temperature. The membranes were then incubated with primary antibodies in 1% non-fat milk at 4 °C overnight (1:1000, primary antibodies are as follows: phospho-ERK (pERK) (Thr 202/Tyr204) (Cell Signaling Technology, Danvers, MA, USA, #4370), p-AKT (S473) (Proteintech Group, Inc., Wuhan, China, 66444-1-Ig), p-GSK-3 β (Tyr 216) (Beyotime Biotechnology, Wuhan, China, #AF1522), p-tau (S404), BACE1 (Abcam, Shanghai, China, #ab92676 and #ab2077), APP (Abcam, Shanghai, China, #ab15272), Ezrin (Abcam, Shanghai, China, #ab75840), Caspase3 (Proteintech, Wuhan, China, #19677-1AP), MAP2 (Affinity, Liyang, China, #AF5156) and GAPDH (GOOD HERE, Hangzhou, China, #AB-P-R001)). The membranes were washed in TBST (5 times, 5 min/time) and incubated with goat anti-rabbit and goat anti-mouse (1:5000, BOSTER Biological Technology Ltd., Wuhan, China, #BA1051 and #BA1054) horseradish, peroxidase-conjugated secondary antibody at 37 °C for 2 h. After washing with TBST (5 times, 5 min/time), the membranes were detected with the enhanced chemiluminescence (ECL) kit (Thermo Fisher Scientific, Waltham, MA, USA). The results were quantified using Bandscan software, v5.0. The quantification of protein was normalized to those of β-actin.

## Data Availability

MS data were deposited to the ProteomeXchange Consortium via the MassIVE with the data set identifiers PXD053527 and PXD053519.

## Supplementary Materials

The following supporting information can be downloaded at: https://www.mdpi.com/article/10.3390/ijms25158336/s1.
