# Supplementary material for: Proteomic Analysis Reveals Physiological Activities of Aβ Peptide for Alzheimer’s Disease
Source: Int J Mol Sci. 2024 Jul 30;25(15):8336. doi: 10.3390/ijms25158336 (PMC11313142; doi:10.3390/ijms25158336)
Supplement: Supplementary file 1 [file ijms-25-08336-s001.zip › Figure S1 multi scatter plot-LFQ-pearsson correlation.pdf]

	1hC1	1hC2	1hC3	1h1n1	1h1n2	1h1n3	1h500n1	1h500n2	1h500n3	1h1u1	1h1u2	1h1u3	1h5u1	1h5u2	1h5u3	4hC1	4hC2	4hC3	4h1n1	4h1n2	4h1n3	4h500n1	4h500n2	4h500n3	4h1u1	4h1u2	4h1u3	4h5u1	4h5u2	4h5u3	12hC1	12hC2	12hC3	12h1n1	12h1n2	12h1n3	12h500n1	12h500n2	12h500n3	12h1u1	12h1u2	12h5u1	12h5u2	24hC1	24hC2	24hC3	24h1n1	24h1n2	24h1n3	24h500n1	24h500n2	24h500n3	24h1u1	24h1u2	24h1u3	24h5u1	24h5u2	24h5u3
1hC1
1hC2	0.99524
1hC3	0.99323	0.99773
1h1n1	0.99852	0.99362	0.99152
1h1n2	0.99861	0.99611	0.99412	0.99823
1h1n3	0.99823	0.99672	0.99641	0.99695	0.99815
1h500n1	0.99778	0.98958	0.98638	0.99758	0.9958	0.99383
1h500n2	0.99742	0.99809	0.99678	0.99576	0.99674	0.99778	0.99293
1h500n3	0.99929	0.99517	0.99454	0.99769	0.9985	0.99893	0.99638	0.99748
1h1u1	0.99822	0.99524	0.99375	0.99917	0.99798	0.99754	0.99686	0.99639	0.9977
1h1u2	0.99845	0.99742	0.9967	0.99585	0.99762	0.99874	0.99394	0.99896	0.99887	0.99634
1h1u3	0.99887	0.99616	0.99591	0.99758	0.99842	0.99872	0.99584	0.99802	0.9995	0.99817	0.99893
1h5u1	0.9986	0.99722	0.99515	0.9984	0.99895	0.99855	0.99581	0.9978	0.99842	0.99882	0.99782	0.99848
1h5u2	0.99701	0.99809	0.99812	0.99629	0.99729	0.99795	0.99215	0.99834	0.99736	0.9974	0.99817	0.99821	0.99809
1h5u3	0.99806	0.99713	0.99692	0.9959	0.99778	0.99918	0.99312	0.99808	0.99893	0.9964	0.9992	0.99885	0.99801	0.99802
4hC1	0.59355	0.62688	0.63994	0.58434	0.59893	0.60733	0.56678	0.61321	0.59914	0.59515	0.61315	0.60602	0.60049	0.62032	0.61344
4hC2	0.59068	0.62328	0.63648	0.58167	0.59611	0.605	0.56401	0.60999	0.59678	0.59218	0.61017	0.60346	0.59754	0.61684	0.61091	0.99904
4hC3	0.60323	0.6367	0.6513	0.59454	0.60945	0.61812	0.57596	0.62368	0.61018	0.60521	0.62344	0.61721	0.61045	0.63127	0.62431	0.99685	0.99734
4h1n1	0.59147	0.62359	0.63657	0.5828	0.59725	0.60441	0.5656	0.60999	0.59704	0.59342	0.61021	0.60408	0.59824	0.61749	0.61065	0.99852	0.99804	0.99732
4h1n2	0.60651	0.63985	0.65388	0.59752	0.61208	0.62044	0.5798	0.62638	0.61265	0.60858	0.62634	0.61994	0.61348	0.63401	0.62675	0.99752	0.99678	0.99862	0.99874
4h1n3	0.5963	0.62934	0.64368	0.58822	0.60188	0.61056	0.5699	0.617	0.60286	0.59879	0.61614	0.61025	0.60335	0.62416	0.6167	0.99537	0.99582	0.99886	0.99718	0.99861
4h500n1	0.58359	0.61524	0.62857	0.57492	0.58881	0.59689	0.55786	0.60229	0.5893	0.58541	0.60238	0.59619	0.59023	0.60948	0.60292	0.99912	0.99947	0.99657	0.99861	0.99669	0.99564
4h500n2	0.59838	0.63156	0.6455	0.58997	0.60405	0.61213	0.57224	0.61847	0.60462	0.60096	0.61792	0.61197	0.60557	0.6261	0.61839	0.99661	0.99652	0.99894	0.99834	0.99939	0.99952	0.99654
4h500n3	0.6149	0.64921	0.66362	0.60651	0.62082	0.62926	0.58809	0.63601	0.62161	0.61774	0.63518	0.62931	0.62254	0.64392	0.63579	0.99456	0.99435	0.99854	0.99617	0.99875	0.99878	0.99386	0.99906
4h1u1	0.59728	0.62922	0.64303	0.58846	0.60284	0.61103	0.57095	0.61588	0.60337	0.59925	0.61638	0.6104	0.60375	0.62345	0.61713	0.99849	0.99884	0.99814	0.99922	0.99861	0.9973	0.99882	0.99814	0.99648
4h1u2	0.60001	0.63338	0.64722	0.59131	0.6057	0.61401	0.5737	0.61983	0.60625	0.60253	0.61952	0.61352	0.60726	0.62744	0.62029	0.99729	0.99695	0.99878	0.99867	0.99956	0.99897	0.99694	0.99966	0.99873	0.9986
4h1u3	0.61754	0.65191	0.66629	0.60921	0.62358	0.63218	0.59017	0.63867	0.62442	0.62036	0.63787	0.63214	0.62511	0.64656	0.63855	0.99334	0.99356	0.99821	0.99519	0.99799	0.9984	0.99264	0.99841	0.99955	0.99588	0.998
4h5u1	0.58572	0.61752	0.63118	0.57738	0.59088	0.59868	0.56011	0.6045	0.59159	0.58806	0.60445	0.59868	0.59242	0.61217	0.60512	0.9973	0.99704	0.99754	0.99918	0.99871	0.99839	0.99777	0.99905	0.99692	0.99852	0.9991	0.99593
4h5u2	0.59235	0.62516	0.63895	0.5834	0.59789	0.60637	0.56581	0.6119	0.59847	0.59411	0.61198	0.6054	0.59925	0.61946	0.61258	0.99915	0.99907	0.99837	0.99857	0.99829	0.99742	0.99902	0.99816	0.99643	0.99896	0.99846	0.99542	0.99835
4h5u3	0.62343	0.65848	0.67324	0.61427	0.629	0.63898	0.59543	0.64527	0.63048	0.62571	0.64463	0.63772	0.63095	0.65292	0.64528	0.99557	0.99518	0.99794	0.99456	0.99741	0.99652	0.99381	0.9969	0.99799	0.99586	0.99702	0.99769	0.99444	0.9966
12hC1	0.55598	0.58424	0.59793	0.54814	0.5604	0.56793	0.53255	0.57145	0.56088	0.55878	0.57243	0.56782	0.56105	0.57932	0.57348	0.98937	0.99088	0.9846	0.98966	0.98492	0.98447	0.99237	0.98545	0.98079	0.9898	0.98585	0.97999	0.98828	0.98862	0.97935
12hC2	0.5467	0.57478	0.58796	0.53862	0.55123	0.5588	0.52344	0.56164	0.55153	0.54936	0.56294	0.55818	0.55191	0.56917	0.56423	0.98796	0.98957	0.98168	0.98798	0.98219	0.98133	0.99116	0.98256	0.97702	0.98794	0.98329	0.97597	0.98634	0.98678	0.97594	0.99927
12hC3	0.55321	0.58139	0.59514	0.54514	0.5577	0.56554	0.52974	0.56845	0.55842	0.55588	0.56971	0.56526	0.55852	0.57605	0.57108	0.98933	0.99126	0.98477	0.98988	0.98494	0.98455	0.99264	0.98544	0.98058	0.99006	0.98593	0.9798	0.98858	0.98873	0.97899	0.99932	0.99946
12h1n1	0.55243	0.58115	0.59447	0.54463	0.55707	0.56449	0.52891	0.5682	0.55738	0.55526	0.56904	0.56424	0.5578	0.57578	0.57012	0.99016	0.99139	0.98501	0.99052	0.98557	0.98512	0.99296	0.98609	0.98115	0.99021	0.98653	0.98019	0.98928	0.98922	0.97967	0.99967	0.99939	0.9993
12h1n2	0.55809	0.58644	0.60026	0.55055	0.5629	0.57008	0.53468	0.57379	0.56336	0.56117	0.57461	0.57049	0.56354	0.5818	0.57578	0.98988	0.99158	0.98659	0.99108	0.98677	0.98691	0.99303	0.98767	0.9834	0.991	0.98773	0.98286	0.99007	0.98964	0.98116	0.99932	0.99858	0.9993	0.99909
12h1n3	0.54457	0.5722	0.58582	0.53716	0.54922	0.5564	0.52186	0.55982	0.5498	0.5476	0.56075	0.55671	0.54999	0.56747	0.56206	0.98776	0.98991	0.98376	0.98917	0.98401	0.98451	0.99172	0.98524	0.98006	0.98884	0.9854	0.97904	0.98859	0.98773	0.9771	0.99882	0.99884	0.99926	0.99904	0.99911
12h500n1	0.56358	0.59448	0.60757	0.55589	0.56837	0.57632	0.53937	0.58165	0.56859	0.56668	0.58119	0.57567	0.56993	0.5885	0.582	0.99262	0.99356	0.98858	0.99221	0.9886	0.98878	0.99462	0.98941	0.98546	0.99186	0.98959	0.98448	0.99134	0.9919	0.98436	0.9984	0.99738	0.9976	0.99879	0.9979	0.99751
12h500n2	0.57664	0.608	0.62135	0.56832	0.5814	0.59006	0.55137	0.59456	0.58185	0.5794	0.59471	0.58884	0.5828	0.60154	0.59585	0.99316	0.99408	0.98848	0.99207	0.98875	0.98758	0.9945	0.98851	0.98521	0.99252	0.98922	0.98456	0.99026	0.9918	0.98565	0.99808	0.99717	0.99742	0.99822	0.99727	0.99621	0.99897
12h500n3	0.54813	0.57602	0.58975	0.5406	0.55272	0.56016	0.52511	0.56364	0.55348	0.55104	0.56452	0.56032	0.55358	0.57126	0.56579	0.98897	0.99104	0.98461	0.98951	0.98441	0.98475	0.99268	0.98546	0.98044	0.98955	0.98566	0.97948	0.98869	0.98875	0.9785	0.99893	0.99906	0.99948	0.99899	0.9992	0.99949	0.99749	0.99655
12h1u1	0.53949	0.56744	0.58065	0.53174	0.544	0.55117	0.51668	0.5546	0.54428	0.54219	0.55556	0.55095	0.54467	0.56218	0.55679	0.98828	0.9897	0.98298	0.98907	0.98351	0.98351	0.99171	0.98443	0.97867	0.9884	0.98486	0.97735	0.98823	0.98765	0.97637	0.99915	0.99929	0.99904	0.99951	0.99856	0.99921	0.99794	0.99657	0.99901
12h1u2	0.55416	0.58263	0.59607	0.54621	0.55857	0.56628	0.53076	0.56965	0.55926	0.5571	0.57061	0.56613	0.55962	0.57732	0.57189	0.98976	0.99135	0.9847	0.99007	0.98509	0.98452	0.99284	0.98564	0.98083	0.9901	0.98619	0.97989	0.98883	0.98894	0.9793	0.99943	0.99951	0.99968	0.99957	0.99917	0.99932	0.99821	0.99793	0.99946	0.99921
12h5u1	0.56219	0.5908	0.60438	0.55415	0.5669	0.57426	0.5386	0.57771	0.56735	0.56499	0.57887	0.57426	0.56746	0.58587	0.58005	0.99108	0.99236	0.98692	0.99175	0.98746	0.98671	0.99371	0.98784	0.98363	0.99172	0.98819	0.98277	0.99046	0.99056	0.98217	0.99935	0.99882	0.99916	0.99932	0.99929	0.99864	0.99806	0.99791	0.99874	0.99864	0.99928
12h5u2	0.54677	0.57518	0.58865	0.53902	0.55137	0.55882	0.52373	0.56245	0.55188	0.54965	0.56328	0.55872	0.55221	0.56986	0.56453	0.9894	0.99084	0.98449	0.99027	0.9852	0.98508	0.99266	0.98591	0.98065	0.98973	0.98634	0.97941	0.98941	0.9888	0.97848	0.99888	0.99915	0.99931	0.99935	0.99886	0.99937	0.99792	0.99694	0.99929	0.99949	0.9994	0.99901
24hC1	0.54773	0.57833	0.59154	0.54	0.5528	0.5617	0.5228	0.56592	0.55318	0.55022	0.56541	0.55981	0.55401	0.57205	0.56659	0.98284	0.98489	0.97961	0.98143	0.97758	0.97912	0.98516	0.97902	0.97465	0.9819	0.97912	0.97458	0.98037	0.98242	0.97451	0.99152	0.99071	0.99087	0.99131	0.991	0.99005	0.99228	0.99117	0.99088	0.99075	0.99081	0.99032	0.99025
24hC2	0.54001	0.56944	0.58213	0.5325	0.545	0.55324	0.51543	0.55713	0.54512	0.54232	0.55693	0.55156	0.54589	0.56326	0.5583	0.98098	0.98322	0.97734	0.98075	0.97608	0.97761	0.98385	0.97755	0.97249	0.98062	0.9776	0.97232	0.97977	0.98059	0.97141	0.99164	0.99113	0.99115	0.99169	0.99129	0.99082	0.99172	0.99041	0.99107	0.99144	0.99091	0.99044	0.99084	0.99912
24hC3	0.52024	0.54864	0.56172	0.51284	0.52499	0.5333	0.49653	0.53697	0.52538	0.52243	0.53676	0.53152	0.52601	0.54296	0.53811	0.97884	0.98121	0.97509	0.97867	0.97332	0.97587	0.98251	0.97554	0.96931	0.97805	0.97545	0.96862	0.97851	0.97885	0.96745	0.99078	0.9909	0.99071	0.99114	0.99037	0.99105	0.99103	0.98824	0.99119	0.99205	0.99039	0.98933	0.99105	0.99802	0.99873
24h1n1	0.54114	0.57044	0.58324	0.53331	0.54603	0.55453	0.51671	0.55781	0.54624	0.54355	0.55798	0.55271	0.54699	0.56409	0.55934	0.98157	0.98371	0.97825	0.98191	0.97744	0.97868	0.98443	0.97879	0.97382	0.98173	0.97904	0.97365	0.98093	0.98112	0.97209	0.99237	0.99191	0.99202	0.99241	0.99207	0.99172	0.99227	0.99106	0.99155	0.99224	0.99182	0.99131	0.99164	0.99878	0.99945	0.99846
24h1n2	0.53704	0.56628	0.57942	0.52957	0.54215	0.55039	0.51263	0.55416	0.54235	0.53949	0.55395	0.54875	0.54307	0.56055	0.55541	0.98089	0.98289	0.97819	0.98115	0.97688	0.97888	0.98371	0.97875	0.97373	0.98066	0.9786	0.97351	0.98074	0.9809	0.9719	0.99131	0.99073	0.99089	0.99145	0.99141	0.99096	0.99163	0.98969	0.99103	0.99145	0.99065	0.99036	0.99085	0.99883	0.99952	0.999	0.99933
24h1n3	0.53678	0.56587	0.57902	0.52908	0.54167	0.55018	0.51257	0.55374	0.5422	0.53932	0.55368	0.54865	0.54275	0.56017	0.5549	0.98028	0.98257	0.97669	0.97964	0.97497	0.97658	0.98336	0.97661	0.97149	0.97982	0.97674	0.97128	0.9788	0.97999	0.97056	0.99094	0.9908	0.9908	0.99092	0.99075	0.99038	0.99076	0.98929	0.99099	0.99087	0.99064	0.98989	0.99041	0.99862	0.99869	0.99828	0.99856	0.99864
24h500n1	0.53198	0.56111	0.57397	0.5248	0.53687	0.54468	0.50837	0.54912	0.53708	0.5348	0.54861	0.5437	0.53789	0.55541	0.54969	0.98073	0.98287	0.97661	0.98011	0.97495	0.97679	0.9841	0.97685	0.97131	0.98003	0.97692	0.97079	0.97942	0.98038	0.96989	0.99216	0.9918	0.99153	0.99227	0.99137	0.99166	0.99233	0.99028	0.99189	0.99248	0.99171	0.99071	0.99165	0.99892	0.99894	0.99896	0.99869	0.99862	0.99862
24h500n2	0.54152	0.57119	0.58422	0.53356	0.54654	0.55528	0.5167	0.55875	0.54688	0.54371	0.55871	0.55321	0.5475	0.56505	0.56007	0.98138	0.98349	0.97771	0.98044	0.97601	0.97734	0.98393	0.97738	0.97248	0.9806	0.97762	0.97233	0.9793	0.98081	0.97211	0.99122	0.99105	0.99086	0.9912	0.99083	0.9901	0.99137	0.99029	0.99068	0.99098	0.99066	0.99016	0.99037	0.99903	0.99871	0.9982	0.99871	0.9986	0.99842	0.99846
24h500n3	0.54295	0.57377	0.58697	0.53594	0.54802	0.55616	0.519	0.56192	0.54837	0.54633	0.5604	0.55546	0.54944	0.56817	0.5611	0.98294	0.98451	0.98043	0.98209	0.97848	0.98088	0.98544	0.98054	0.97611	0.98201	0.98033	0.97578	0.98188	0.98292	0.97494	0.99099	0.98971	0.99004	0.99113	0.99078	0.99001	0.99267	0.99041	0.99061	0.99086	0.99037	0.98993	0.99041	0.99809	0.99727	0.99699	0.99684	0.99735	0.99828	0.99817	0.99686
24h1u1	0.5551	0.58557	0.5986	0.54696	0.56021	0.56951	0.52928	0.57315	0.56058	0.55708	0.57299	0.567	0.56127	0.57903	0.57442	0.98327	0.98544	0.98011	0.98222	0.97861	0.97957	0.98533	0.9795	0.97551	0.98268	0.97972	0.97557	0.98071	0.98254	0.97563	0.99092	0.9902	0.99055	0.99077	0.99074	0.98949	0.99147	0.99119	0.99005	0.98974	0.99017	0.99002	0.98969	0.99892	0.99901	0.99696	0.99877	0.99856	0.99787	0.99736	0.99837	0.99606
24h1u2	0.54874	0.57897	0.59206	0.54044	0.5536	0.56317	0.5232	0.56651	0.55429	0.55077	0.56651	0.56052	0.55507	0.57254	0.56798	0.98299	0.98526	0.97946	0.9817	0.97776	0.97887	0.98533	0.97887	0.9745	0.98211	0.97917	0.97434	0.98043	0.98239	0.9746	0.99139	0.99113	0.99134	0.99132	0.99117	0.99037	0.99197	0.99142	0.9911	0.99071	0.99105	0.99046	0.99055	0.99906	0.99892	0.99777	0.99886	0.99873	0.99843	0.99778	0.9987	0.99641	0.99947
24h1u3	0.55862	0.58998	0.60332	0.55041	0.56376	0.57335	0.53269	0.57744	0.56429	0.5609	0.57701	0.5708	0.56524	0.5835	0.57826	0.98439	0.9861	0.98077	0.98223	0.97892	0.97967	0.9859	0.9797	0.97596	0.98292	0.97997	0.97593	0.98072	0.98348	0.97717	0.99056	0.98987	0.99004	0.99046	0.99018	0.98859	0.99176	0.9916	0.98973	0.98919	0.98992	0.98973	0.98912	0.99916	0.99839	0.99643	0.99788	0.998	0.99778	0.99719	0.99829	0.99673	0.99936	0.99932
24h5u1	0.53342	0.56184	0.57421	0.52558	0.5384	0.54667	0.50919	0.54941	0.53842	0.53534	0.54992	0.54455	0.53893	0.55533	0.55141	0.97758	0.98035	0.97273	0.97722	0.97153	0.9725	0.98099	0.97266	0.9669	0.97745	0.97301	0.96672	0.97549	0.97677	0.96619	0.9903	0.99066	0.99033	0.99025	0.98967	0.98966	0.98911	0.98833	0.9899	0.99016	0.98985	0.98921	0.9897	0.99796	0.999	0.99787	0.99888	0.99826	0.99819	0.99817	0.99798	0.99509	0.99825	0.99832	0.99727
24h5u2	0.5339	0.56291	0.57612	0.5266	0.53877	0.54661	0.51051	0.55089	0.5392	0.5368	0.55057	0.54589	0.53975	0.55756	0.55158	0.98155	0.98342	0.97741	0.98101	0.97604	0.97766	0.98478	0.97772	0.97231	0.98089	0.97779	0.97174	0.98033	0.98124	0.97112	0.99233	0.99208	0.99194	0.9925	0.99187	0.99175	0.9923	0.99038	0.99231	0.99269	0.992	0.99137	0.99219	0.9986	0.99861	0.99866	0.99831	0.99841	0.99865	0.99949	0.99855	0.99832	0.99699	0.99755	0.9972	0.99784
24h5u3	0.55688	0.59006	0.60275	0.54971	0.56243	0.57065	0.53152	0.57736	0.5622	0.55974	0.57549	0.56952	0.5641	0.58352	0.57657	0.98586	0.98683	0.98345	0.98425	0.98179	0.9839	0.98715	0.98332	0.97969	0.98379	0.98284	0.97928	0.98421	0.98599	0.98011	0.98817	0.98662	0.98731	0.98872	0.98837	0.98677	0.99138	0.9896	0.98769	0.98771	0.9872	0.9879	0.98767	0.9946	0.99414	0.99283	0.99261	0.99392	0.99245	0.99326	0.99328	0.99479	0.99403	0.99332	0.99495	0.99068	0.99369
